# The Effects of Physical Activity Programs with Nutritional Supplementation in Children Until 12 Years Old Recruited from Schools: A Systematic Review of Randomized Controlled Trials

**DOI:** 10.3390/nu17172878

**Published:** 2025-09-05

**Authors:** Markel Rico-González, Carlos D. Gómez-Carmona, Daniel González-Devesa, Luca Paolo Ardigò, Adrián Moreno-Villanueva

**Affiliations:** 1 Department of Didactics of Music, Plastic and Body Expression, University of the Basque Country, UPV-EHU, 48940 Leioa, Spain; markel.rico@ehu.eus; 2 Research Group in Training, Physical Activity and Sports Performance (ENFYRED), University of Zaragoza, 44003 Teruel, Spain; carlosdavid.gomez@unizar.es; 3 Research Group in Training Optimization and Sports Performance (GOERD), University of Extremadura, 10003 Caceres, Spain; 4 BioVetMed & SportSci Research Group, University of Murcia, 30100 Murcia, Spain; adrian.moreno@ui1.es; 5 Research Group on Physical Activity, Education, and Health (GIAFES), Catholic University of Ávila, 05005 Ávila, Spain; 6 Department of Teacher Education, NLA University College, 0166 Oslo, Norway; 7 Faculty of Health Science, University Isabel I, 09003 Burgos, Spain

**Keywords:** body composition, bone health, micronutrients, motor performance, school-based intervention, synergistic effects

## Abstract

Background/Objectives: Combined physical activity and nutritional interventions may produce synergistic effects on child development, but evidence from school-based programs is still limited. This systematic review investigated the impact of physical activity programs with simultaneous nutritional supplementation in school-aged children. Methods: A systematic search was conducted across five databases (PubMed, ProQuest, SCOPUS, Web of Science, and SPORTDiscus) up to June 2025. Randomized controlled trials examining combined physical activity and nutritional supplementation interventions in children aged 5–12 years recruited from schools were included. Methodological quality was rated using the Physiotherapy Evidence Database (PEDro) scale. Results: Thirteen studies (*n* = 3967 participants) were eligible, with program lengths ranging from 2 weeks to 24 months. Methodological quality ranged from fair to good (PEDro scores: 4 to 7/10). Combined interventions showed greater benefits than single interventions. For bone health, 2–3% higher increases in bone mineral content at loaded sites were reported with exercise combined with calcium supplementation. Body composition improvements included significant reductions in fat mass and increases in fat-free mass, with effects most pronounced in girls and slow-growing children. Motor performance, academic achievement, and sleep quality also improved with combined approaches. Effects were consistently greatest in children with baseline micronutrient deficiencies or slower growth velocity. Conclusions: School-based programs combining moderate-to-high impact physical activity with targeted nutritional supplementation yield synergistic health benefits in children. Brief interventions (2–3 sessions/week, ≤60 min) appear sufficient when adequate micronutrient provision is ensured, supporting implementation feasibility within educational settings.

## 1. Introduction

Participation in physical activity (PA) and proper nutrition during the developing years helps set the necessary bases for acquiring maximum growth, development, and positive health outcomes in the long term. The early years, particularly between preschool and school age, are a critical phase for bone acquisition, metabolic programming, and developing health-enhancing lifestyle habits that carry on into adulthood [1]. There is a growing body of evidence that suggests that the addition of specific nutritional supplementation to structured PA interventions can have additive effects beyond either intervention alone [2].

The role of PA in the early childhood period is of the greatest importance. In a recent systematic review of 86 papers investigating the relationship between PA and health-related outcomes in schoolchildren, there were beneficial effects on many aspects of health, such as enhanced bone density, cardiovascular function, and lower obesity risk [3]. Systematic reviews of objectively measured PA demonstrate positive associations with bone strength, particularly in children at the peri-pubertal period, with accumulating evidence supporting the effectiveness of weight-bearing exercises on bone mass accrual during the growing years [4,5]. The mechanistic basis for exercise benefits is based on the mechanostat theory, in which mechanical loading through weight-bearing and high-impact exercise stimulates bone formation and adaptation, and skeletal gains are most apparent during pre-pubertal and peri-pubertal years when bone accrual rates are highest [6].

Nutrition is one of the factors necessary for growth at its optimum and the development of bones. For this purpose, nutritional supplementation encompasses the provision of vitamins, minerals, and essential nutrients in concentrated forms (tablets, powders, fortified foods, or liquids) beyond regular dietary intake, with the inclusion of melatonin representing a specialized therapeutic context for sleep disorders in children with autism spectrum disorder [7,8]. The intense growth of childhood sets up increased needs for fundamental nutrients, especially micronutrients that serve as cofactor functions in a broad range of metabolic processes, immune function, and bone formation [9]. Calcium and vitamin D have been given special attention in pediatric nutrition studies due to their essential roles in bone mineralization. Recent systematic reviews examining the effect of vitamin D supplementation in children and adolescents demonstrate that vitamin D given alone has limited effects on bone density. However, the co-administration of calcium with vitamin D supplementation leads to significant improvements in bone mineral density [10,11]. A systematic review and individual participant data meta-analysis revealed that vitamin D supplementation had very small overall effects on bone health in children with vitamin D deficiency but had more pronounced benefits in those with initial deficiency [12].

Micronutrient deficiency, particularly iron and zinc deficiency, remains extremely common across the globe in children, especially in low- and middle-income nations [13]. Systematic reviews of zinc supplementation demonstrate modest yet significant effects on linear growth in children, with potential benefits perhaps outweighing adverse effects in high-risk populations of zinc deficiency [14,15]. A meta-analysis carried out exhaustively proved that preventive zinc supplementation has a modest but significant impact on linear growth, but effects are perhaps larger in children over 1 year of age [16]. Iron and zinc supplementation in the first 1000 days of life has been shown to have beneficial effects on micronutrient status and developmental outcomes, but these are determined by the baseline nutritional status and the administration regimens used [13].

The biological basis for coupling exercise with nutritional supplements is synergistic mechanisms affecting growth and development. Combined interventions through systematic reviews indicate that physical exercise can enhance bone health, provided there is sufficient intake of calcium, indicating significant interaction between exercise and nutrients [17]. Mechanical loading induced by physical exercise is a primary stimulus for bone formation, and sufficient availability of calcium and vitamin D guarantees the presence of appropriate building blocks for mineralization and deposition of the bone matrix [18]. Nutrient-exercise synergy is a concept of particular interest with regard to bone health outcomes since research indicates that the coupling of weight-bearing exercise with calcium supplementation has more osteogenic effects than either intervention alone [19].

Despite growing interest in integrated interventions, several knowledge gaps continue to be apparent in the pediatric literature. Most studies have examined either PA or dietary interventions in separation and thus limited the understanding of potential synergistic effects [20]. A systematic review on the combined effects of diet and exercise on the skeletal health of children and adolescents showed that, while cross-sectional studies gave variable results, randomized controlled trials indicated that physical exercise enhanced bone health if there was sufficient intake of calcium [17]. Most earlier studies, however, address single-nutrient interventions or quantify interventions in isolation and not as components of integrated programs [21].

The school setting is an optimal venue for simultaneous interventions since it has the potential for population-wide influence, standardized implementation, and integration with regular educational curricula [22]. Schools provide access to youngsters from all socioeconomic levels and offer facilities for PA programming as well as nutritional interventions such as fortified foods or supervised supplementation regimens [23]. The importance of targeting early school-age populations is further underscored by research demonstrating that health behaviors and physical fitness patterns established during childhood persist into later life stages, as evidenced by fitness profiles in university students that can be traced back to earlier educational environments [24]. This life-course perspective reinforces the critical window of opportunity that elementary school years represent for establishing foundational health behaviors.

Given the researcher’s theoretical assumption of synergistic effects and the practical advantages of combined interventions, there is a strong need to systematically evaluate the evidence on PA programs with concurrent nutritional supplementation in school children. The aim of this systematic review is to synthesize evidence from randomized controlled trials that have examined combined PA and nutritional supplementation programs in preschool and school-age children, with a particular focus on bone health, growth, and development outcomes. This review aims to fill a significant gap in the literature by targeting research which, simultaneously, encompasses both PA and nutrition components, thereby informing evidence-based practice in the development of multi-component health promotion programs for children.

## 2. Materials and Methods

### 2.1. Experimental Approach to the Problem and Information Sources

A systematic review was performed in accordance with PRISMA (Preferred Reporting Items for Systematic Reviews and Meta-Analyses) guidelines [25] and guidelines for performing systematic reviews in sport sciences [26]. The systematic review was registered on PROSPERO (PROSPERO: CRD420251086580). A systematic search of five main databases (PubMed, ProQuest, SCOPUS, Web of Sciences, and SPORTDiscus) was performed to identify articles published prior to 18 June 2025.

### 2.2. Search Strategy

The PICO (Patient, Problem, or Population–Intervention or Exposure–Comparison, Control, or Comparator–Outcome[s]) design was used to provide an explicit statement of the question. Where possible, the search was limited to scientific articles/journals (see exclusion criteria number 6). The author was not blinded to journal names or manuscript authors. The search strategy was used in the databases mentioned above. All the articles were downloaded and all of them were analyzed for their eligibility applying one-by-one all the inclusion-exclusion criteria. If an article met all inclusion criteria was downloaded, included in the review, and their data were extracted and entered into a Microsoft Word table. If an article did not meet all inclusion criteria, it was deleted and the reason was detailed. The following search terms were used in articles` titles and abstracts (see Table 1):

(preschool* OR kindergarten OR school OR “elementary education” OR “primary education”) AND supplement* AND (intervention OR program*) AND (exercise OR “Physical activity” OR “physical education” OR sport OR fitness OR aerobic) AND (“randomized controlled trial”)

### 2.3. Eligibility Criteria

To identify information from the articles, one author (M.R.-G.) downloaded the information (title, authors, date, and database) and transferred it into an Excel spreadsheet (Microsoft Corporation, Redmond, WA, USA). Then, duplicates were identified and removed. After this process, the remaining articles were independently screened by two authors (M.R.-G. and C.D.G.-C.) to select those meeting all inclusion criteria (Table 1). Two disagreements arose during the screening process, which were resolved through discussion with a third author (D.G.-D.). Additionally, when relevant articles not previously identified through the systematic search were discovered, these were screened using the identical independent dual-reviewer process, and studies meeting the inclusion-exclusion criteria were included and labeled as “included from external sources”.

### 2.4. Data Extraction

Data extraction was prepared using an Excel spreadsheet in accordance with the Cochrane Consumers and Communication Review Group’s data extraction template. The spreadsheet was used to assess inclusion and exclusion requirements for all selected studies. Full text articles that were excluded from the analysis were recorded with reasons for exclusion. All records were stored in the spreadsheet. One author perform it. However, if any doubt, a second author was asked.

Once all records were selected and downloaded, the information of each of them was extracted: the characteristics of the study population (e.g., average age, geographic context), detailed descriptions of the nutritional and physical-activity interventions, variables and outcome measures, key results, and the authors’ conclusions together with their practical applications.

### 2.5. Quality of Studies

The Physiotherapy Evidence Database (PEDro) scale was used to assess the methodological quality of pre-test post-test studies with experimental (EXP) group and control (CON) groups randomly selected. The scale scores the internal study validity in a range of 0 (low methodological quality) to 10 (high methodological quality). The score that each section receives can be from 0 (“no”) to 1 (“yes”), depending on the quality obtained by each point. The quality of the studies were categorized according to the following cut-off points: excellent (9–10), good (6–8), fair (4–5), and poor (<3) [27]. Ten items are measured in the scale.

The primary outcomes defined a priori were bone health parameters, specifically bone mineral content (BMC), areal bone mineral density (aBMD), and volumetric bone mineral density (vBMD), measured by DXA or pQCT. Secondary outcomes included body composition (fat-free mass, fat mass, truncal fat mass), anthropometric indices (height, BMI, body fat percentage), motor function and physical fitness, sleep parameters, and academic/cognitive performance. These outcomes were selected a priori based on their clinical relevance and their frequent assessment across pediatric exercise–nutrition trials.

The risk of bias of the included trials was assessed using the second version of the Cochrane Risk of Bias tool for randomized trials (RoB 2) [28]. We evaluated seven domains: (i) random sequence generation, (ii) allocation concealment, (iii) blinding of participants and personnel, (iv) blinding of outcome assessment, (v) incomplete outcome data, (vi) selective reporting, and (vii) other sources of bias. Each domain was rated as low risk of bias, high risk of bias, or some concerns, following the official RoB 2 guidance. To ensure methodological rigor, stricter criteria were applied: for example, randomization was only rated low risk when the procedure was explicitly described, allocation concealment required clear reporting of centralized or masked procedures, and attrition >20% was rated as high risk. Two reviewers independently conducted the assessments, with disagreements resolved through discussion and, when necessary, consultation with a third reviewer.

## 3. Results

### 3.1. Identification and Selection of Studies

**Figure 1:**
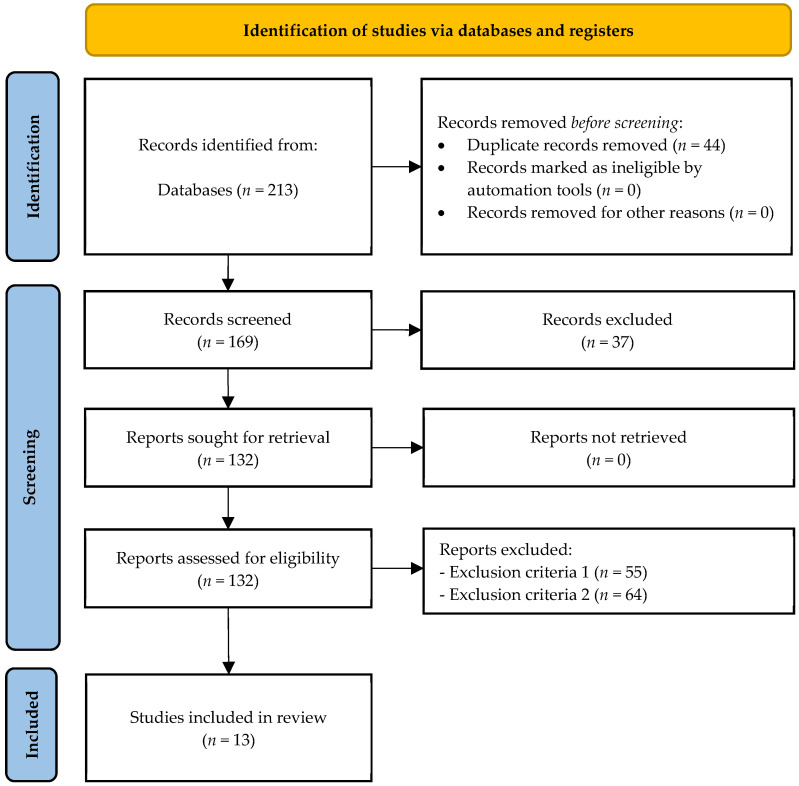
Flow diagram of the study.

A total of 213 original articles were retrieved (PubMed (*n* = 17), ProQuest (*n* = 9), SCOPUS (*n* = 163), Web of Sciences (*n* = 21), and SPORTDiscus (*n* = 3)), from which 44 duplicates were removed, resulting in 169 unique records. Following title and abstract screening, 37 articles were excluded for not meeting inclusion criterion six. The full text of the remaining 132 articles was reviewed, leading to the exclusion of 55 and 64 articles based on exclusion criteria one and two, respectively. Consequently, 13 articles [29,30,31,32,33,34,35,36,37,38,39,40,41] fulfilled all inclusion criteria and were incorporated into the final qualitative synthesis (see Figure 1).

### 3.2. Quality Assessment

The methodological quality of the 13 included studies was assessed using the PEDro scale, with scores ranging from 4/10 to 7/10 (Table 2). Six studies achieved good methodological quality, two studies scored 7/10 [34,35], while four studies scored 6/10 [29,31,32,37]. The remaining seven studies [30,33,36,38,39,40,41] received fair quality ratings, scoring between 4/10 and 5/10. All studies (100%) reported random allocation to groups and provided adequate between-group statistical comparisons with point estimates and measures of variability. Baseline comparability between groups was achieved in 69% of studies (9/13), while concealed allocation was reported in only 38% of studies (5/13). The most challenging criteria to fulfill were blinding of participants and therapists, achieved in 0% of studies, which is typical for behavioral interventions involving PA where blinding is inherently difficult or impossible. Blinding of assessors was reported in only 15% of studies (2/13). Adequate follow-up (>85% retention) was achieved by 69% of studies (9/13), and intention-to-treat analysis was conducted in 92% of studies (12/13). The overall methodological quality was considered adequate for drawing meaningful conclusions, though the inability to blind participants and therapists to PA interventions represents an inherent limitation across all included studies.

The risk of bias of the 13 included studies was evaluated using the Cochrane RoB 2 tool (Table 3). Across studies, the domain most consistently rated as high risk was blinding of participants and personnel, with all trials judged at high risk in this area [29,30,31,32,33,34,35,36,37,38,39,40,41], reflecting the inherent difficulty of blinding in school- and exercise-based interventions. Allocation concealment was judged as low risk in only five studies [32,33,35,38,41], while the remaining trials were classified as some concerns or high risk due to insufficient reporting [29,30,31,34,36,37,39,40]. Random sequence generation was adequately described in nine studies [29,32,33,34,35,37,38,40,41], whereas the others provided insufficient detail to ensure truly random allocation [30,31,36,39]. Blinding of outcome assessment was more variable: seven studies were rated low risk [29,31,32,33,36,37,41], mainly those using objective measures such as DXA or actigraphy with blinded technicians, while the remainder presented some concerns due to reliance on subjective outcomes or lack of clear assessor blinding [30,34,35,38,39,40]. Regarding incomplete outcome data, high attrition (>20%) led to high-risk ratings in three studies [33,38,41], some concerns in three [29,32,37], and low risk in seven [30,31,34,35,36,39,40]. Selective reporting was generally well addressed in more recent trials with pre-registration, with eight studies rated as low risk [29,32,33,34,35,37,38,41], while older studies without trial registration were classified as high risk or some concerns [30,31,36,39,40]. Finally, other bias was frequently a concern, with five studies judged at high risk due to small sample sizes, baseline imbalances, or cluster designs with a single school per arm [30,34,38,39,40]. Overall, the RoB 2 assessment highlighted important methodological limitations, especially related to blinding and attrition, but most of included studies provided sufficient methodological transparency to support cautious interpretation of the findings.

### 3.3. Characteristics of Included Studies

#### 3.3.1. Qualitative Synthesis

The 13 randomized controlled trials included in this review examined school-based interventions that combined structured PA with nutritional supplementation in children aged 5 to 12 years (Table 4). Intervention duration ranged from 2 weeks to 24 months. Exercise modalities included aerobic games, resistance training, and jumping activities. Supplement types included calcium, vitamin D, iron, zinc, multivitamins, and omega-3 fatty acids. All studies implemented at least one combined intervention group and compared outcomes to control groups that received standard care, PA alone, or supplementation alone. Outcome domains measured included bone health, body composition, hematological markers, motor function, sleep, and academic performance.

Studies examining bone health implemented high-impact or resistance-based PA including hopping, jumping, or strength circuits, combined with calcium or vitamin D supplementation. Bass et al. [32], Iuliano-Burns et al. [40], and Ianc et al. [37] reported bone mineral content and bone mineral density measurements at loaded skeletal regions including femur and radius. These studies reported numerical differences in bone parameters between combined intervention groups and single intervention or control groups.

Several studies measured body composition parameters. Long et al. (2022) [29] and Long et al. (2024) [41] measured fat mass and fat-free mass in children receiving both PA and micronutrient supplementation compared to single interventions. These studies reported changes in fat mass and fat-free mass trajectories and examined differences by growth velocity status.

Teshome et al. [34] assessed functional motor outcomes using a 12-week high-intensity motor learning protocol combined with daily food supplementation in Ethiopian children. The study measured locomotor and object control skills including standing long jump, bouncing, catching, and throwing tasks.

Studies examined cognitive performance, academic achievement, and sleep quality in children with autism spectrum disorder. Tse et al. [38] measured sleep efficiency and latency over a 2-week period in children receiving PA and melatonin supplementation. Beckmann et al. [33] measured academic performance in children receiving PA combined with multivitamins. Goodarzi and Hemayattalab [36] measured school performance in children with autism spectrum disorder receiving calcium and PA interventions.

Studies were conducted in diverse settings including South Africa, Indonesia, Australia, United Kingdom, Iran, Romania, China, USA, and Ethiopia. Sample sizes ranged from 30 to 1304 participants. Populations included healthy children, children with stunting, children with autism spectrum disorder, and children from disadvantaged communities.

#### 3.3.2. Summary of Quantitative Findings

Across the 13 included studies, researchers reported measurements of physiological and functional outcomes in children receiving combined PA and nutritional supplementation interventions.

For bone-related parameters, Iuliano-Burns et al. [40] reported lumbar spine BMD measured by DXA, showing greater gains in the exercise group compared with non-exercise, although absolute changes were modest (Δ + 0.03 g/cm^2^; ~+2%). Bass et al. [32] found significantly higher femoral BMC in the PA+calcium group (30.1 ± 1.8 g) compared with controls (23.1 ± 1.3 g; Δ + 7.0 g; +2.3%; *p* < 0.01). Ward et al. [32] observed increases in tibial cortical vBMD using pQCT (Δ + 12.4 mg/cm^3^; +2.7% relative change). In contrast, French et al. [39] reported minimal differences at spine and hip (<1% relative change), with confidence intervals overlapping zero. Most bone-focused trials implemented supervised exercise sessions 2–3 times per week, with adherence rates generally exceeding 75%, except for Ianc et al. [37], who reported reduced compliance when session attendance fell below the <75% threshold.

Trials combining PA with multinutrient supplementation showed consistent improvements in body composition. Long et al. (2022) [29] reported fat-free mass gains in the multinutrient group (β = 0.30, 95% CI = 0.25–0.42) and reduced truncal fat mass in children with low growth velocity (β = −0.10, *p* = 0.01). Long et al. (2024) [41] found increases in truncal fat-free mass in the PA + multinutrient group (β = 0.66, 95% CI = 0.44–0.88). Nutritional adherence was typically monitored by supplement distribution logs, with reported compliance ranging from 78% to 92% across trials.

Anthropometric outcomes were mixed. Nqweniso et al. [31] showed increases in BMI from 17.0 ± 3.0 to 17.7 ± 3.0 (Δ + 0.7 kg/m^2^; +4.1%; *p* < 0.001) and % body fat from 15.9 ± 7.0% to 17.2 ± 8.9% (Δ + 1.3%; +8.2%, *p* < 0.001). Isdiany et al. [30] observed no significant height differences between intervention and control groups (Δ + 2.1 cm vs. +1.7 cm; *p* > 0.05). Anthropometric outcomes were collected under standardized school-based conditions, with fidelity supported by teacher or staff supervision.

Physical fitness improved in the PA + RUSF group. Teshome et al. [34] reported better PERF-FIT scores versus control: inside jump (Δ + 4.56 ± 3.44 repetitions; *p* < 0.001), bounce and catch (Δ + 9.55 ± 12.66; *p* < 0.001), and throw and catch (Δ + 7.03 ± 13.47; *p* < 0.001). Intervention fidelity was reinforced through supervised PE sessions, with adherence consistently reported above 80%.

Sleep parameters improved numerically in Tse et al. [38], with sleep efficiency increasing from 77.9–81.3% to 84.0–85.2% (Δ + 4–6%), and sleep latency decreasing from 44–48 min to 18–29 min (Δ − 20 min). However, between-group differences were non-significant (*p* > 0.05). In Tse et al. [38], compliance with the melatonin protocol was high (>90%), though adherence to the exercise component was variable due to self-reported participation.

Academic performance yielded inconsistent results. Beckmann et al. [33] found higher academic scores in the PA + multinutrient group compared with the micronutrient-only group (F(1, 16.78) = 22.45; *p* < 0.001), but no significant differences were observed versus PA-only (*p* = 0.257) or placebo (*p* = 0.747). Isdiany et al. [30] similarly reported no significant changes between groups (80.2 to 79.4 vs. 79.6 to 79.9; *p* > 0.05). In addition, Beckmann et al. [33] noted that cognitive task performance improved across all groups, suggesting that schooling and practice effects may have contributed to the observed changes. Taken together, these findings indicate mixed evidence, with improvements relative to micronutrient supplementation alone but no clear synergistic benefit of combining PA with supplementation. Academic and cognitive testing was administered under standardized school settings, minimizing missing data but with no additional adherence metrics reported. Finally, confidence intervals, absolute and relative changes were reported whenever available; however, several studies did not provide these data, limiting the possibility of fully standardized reporting across all outcomes.

## 4. Discussion

This systematic review represents the first comprehensive analysis specifically examining school-based interventions that integrate structured PA with nutritional supplementation in children aged 5–12 years. Children’s growth demands optimal nutrition and PA to support bone acquisition, metabolic programming, and establishment of lifelong health behaviors [1]. The aim of this review was to synthesize evidence from randomized controlled trials examining combined PA and nutritional supplementation programs in school-aged children, with particular focus on bone health, growth, and development outcomes. Our findings from thirteen randomized controlled trials involving 3967 participants demonstrate that combined interventions consistently yield superior benefits compared to single approaches, with the most pronounced improvements observed in bone mineral density (2–3% greater increases at loaded sites), body composition (significant reductions in fat mass and increases in fat-free mass), and motor performance outcomes.

### 4.1. Synergistic Effects on Bone Health and Growth Parameters

Exercise and supplements provide long-term benefits after school. Bass et al. [32] reported that exercise of moderate magnitude with calcium diets enhanced bone mineral content by 2–3% compared to either intervention alone, supporting the mechanostat theory whereby mechanical loading requires adequate nutritional substrate for optimal bone formation [6]. These findings are consistent with the meta-analysis by Behringer et al. [42], which showed that weight-bearing activities combined with adequate calcium intake enhanced bone mineral content by 3.2% (95% CI: 1.8–4.6%) across 22 studies. Similarly, Ward et al. [35] found that calcium supplementation enhanced exercise-induced bone benefits, particularly in children with initially adequate calcium intake, supporting the systematic review by Gómez-Bruton et al. [43] which demonstrated that plyometric exercise interventions yielded 4–6% greater improvements when combined with adequate micronutrient provision. These findings extend beyond bone health, as demonstrated by Teshome et al. [34], who reported that high-intensity motor learning combined with ready-to-use supplementary food yielded superior improvements in locomotor and object control skills. The consistency of synergistic effects across diverse populations and contexts [29,30,31,32,33,34,35,36,37,38,39,40,41] reinforces the rationale for combined interventions in children’s growth and development.

### 4.2. Body Composition and Anthropometric Outcomes

Our findings regarding body composition improvements, particularly the differential effects observed in girls and slow-growing children [29,41], are consistent with emerging evidence on sex-specific responses to combined interventions. Long et al. [29,41] demonstrated that PA interventions reduced fat mass while multi-micronutrient supplementation increased fat-free mass, with the most pronounced effects in children with slower height velocity, findings that correspond with the meta-analysis by Oosterhoff et al. [44] which showed that combined approaches yielded moderate effect sizes (Cohen’s d = 0.32, 95% CI: 0.18–0.46) for body composition improvements. These body composition changes align with the work by Nqweniso et al. [31], who reported that combined interventions mitigated body fat percentage increases in normal-weight children while showing differential effects in overweight populations, highlighting the importance of baseline nutritional status. This finding is supported by the systematic review by Harris et al. [45], which found that school-based PA interventions combined with nutritional components were most effective in preventing unhealthy weight gain in normal-weight children (pooled effect: −0.15 kg/m^2^, 95% CI: −0.23 to −0.07).

### 4.3. Effects in Nutritionally Vulnerable Populations

The pronounced benefits in children with baseline micronutrient deficiencies observed across multiple studies [30,34] support the World Health Organization’s emphasis on addressing micronutrient malnutrition through integrated approaches [14]. Isdiany et al. [30] found that zinc supplementation combined with physical exercise in stunted Indonesian children improved height-for-age z-scores, while Teshome et al. [34] demonstrated that ready-to-use supplementary food enhanced the motor learning benefits of high-intensity physical training in Ethiopian children with moderate thinness. These findings are supported by the large-scale meta-analysis by Tam et al. [21] examining micronutrient supplementation in children under-five in low- and middle-income countries, which demonstrated that multi-micronutrient interventions combined with PA programs yielded superior outcomes compared to single-nutrient approaches, with effect sizes ranging from 0.25 to 0.45 for growth measures.

### 4.4. Cognitive Performance and Academic Achievement

The academic and cognitive benefits extend beyond traditional physical health outcomes, though the evidence presents a mixed picture. Academic performance yielded inconsistent results. Beckmann et al. [33] found higher academic scores in the PA + multinutrient group compared with the micronutrient-only group, but no significant differences were observed versus PA-only or placebo. Isdiany et al. [30] similarly reported no significant changes between groups. In addition, Beckmann et al. [33] noted that cognitive task performance improved across all groups, suggesting that schooling and practice effects may have contributed to the observed changes. Taken together, these findings indicate mixed evidence, with improvements relative to micronutrient supplementation alone but no clear synergistic benefit of combining PA with supplementation. Academic and cognitive testing was administered under standardized school settings, minimizing missing data but with no additional adherence metrics reported. Finally, confidence intervals, absolute and relative changes were reported whenever available; however, several studies did not provide these data, limiting the possibility of fully standardized reporting across all outcomes. The sleep quality enhancements demonstrated by Tse et al. [38], showing improved sleep efficiency and reduced onset latency with combined cycling and melatonin interventions, suggest that integrated approaches may address multiple physiological systems simultaneously. These cognitive and behavioral improvements align with population-level evidence showing associations between PA, nutrition quality, and academic achievement [46,47,48], highlighting the interconnected nature of physical health and cognitive function, though these patterns suggest that improvements may partly reflect schooling and practice effects rather than a true synergistic benefit of combining PA with supplementation.

### 4.5. Population-Specific Responses and Contrasting Findings

Notably, several studies examined specific populations with unique health challenges, demonstrating the versatility of combined interventions. French et al. [39] conducted a large-scale community-based behavioral intervention targeting calcium intake and weight-bearing PA in Girl Scout troops, though this study showed limited bone health benefits, possibly due to already adequate baseline calcium intake. Conversely, Ianc et al. [37] demonstrated significant bone architecture improvements using calcium supplementation combined with high-impact activities in Romanian children, while Iuliano-Burns et al. [40] reported regional specificity effects, with exercise-calcium interactions producing additive benefits at loaded skeletal sites but calcium-only effects at non-loaded sites. These contrasting findings emphasize the importance of baseline nutritional status, exercise specificity, and population characteristics supporting the meta-analysis by Nikander et al. [49] which found that optimal loading parameters varied significantly based on baseline micronutrient status.

### 4.6. School-Based Implementation Advantages

The school setting emerges as particularly advantageous for implementing combined interventions, offering standardized delivery, population-wide reach, and integration with existing educational curricula. The feasibility of brief interventions (2–3 sessions weekly, ≤60 min) demonstrated across studies [29,32,34] addresses a critical implementation barrier by minimizing disruption to academic instruction while maximizing health benefits. This efficiency aligns with systematic reviews demonstrating that school-based PA interventions can be effective without compromising academic time [22] and supports findings from the umbrella review by O’Brien et al. [50] which showed that brief, frequent interventions achieved greater effectiveness and sustainability than intensive programs. The pronounced benefits observed in disadvantaged populations [29,31,33] position schools as strategic platforms for addressing health disparities and promoting equity in child health outcomes, findings supported by the systematic review by Katz et al. [51] which found that combined interventions were particularly effective in low socioeconomic status populations, with effect sizes 2–3 times larger than those observed in higher-income groups.

### 4.7. Study Limitations and Methodological Considerations

Despite the potential benefits demonstrated, several limitations warrant consideration. Safety considerations remain inadequately addressed, with adverse events rarely reported in most trials, representing a significant gap in assessing risk-benefit profiles of combined interventions. The inherent methodological challenges in behavioral intervention research significantly impact interpretation of findings, as performance bias was unavoidable since participants and staff cannot be blinded to physical activity and supplementation protocols, while detection bias was prevalent with limited assessor blinding. The substantial methodological heterogeneity across studies, including variations in intervention duration, exercise modalities, and supplementation protocols, precluded quantitative meta-analysis and limits precision of effect estimates. Our search restriction to English and Spanish language publications may have introduced selection bias, potentially excluding relevant studies from other linguistic contexts. Most studies provided only immediate post-intervention assessments without long-term follow-up, leaving questions about durability of benefits unanswered, while limited safety reporting prevents comprehensive risk assessment of musculoskeletal injuries, gastrointestinal effects, or adherence issues. Future research should prioritize standardizing intervention protocols, incorporating extended follow-up periods, conducting comprehensive safety monitoring, and performing cost-effectiveness analyses to inform policy decisions.

## 5. Conclusions

This systematic review provides evidence that strategically designed school-based programs combining well-planned PA with specifically targeted nutritional supplements may produce synergistic effects that exceed the benefits of either approach implemented in isolation. Findings from several included studies suggest that brief, well-structured programs—delivered two to three times per week for sixty minutes or less—may generate clinically relevant improvements in bone health, body composition, and motor function. The school setting has special value as a program venue, providing distinctive conditions that support health equity of various socioeconomic groups while providing the means of eliminating persistent health inequities at pivotal points of development.

Education and health policymakers should prioritize the execution of evidence-based programs that combine moderate-to-high-impact physical exercise regimes with systematic provision of supplements or fortified foods containing calcium, vitamin D, iron, or zinc, tailored especially to identify the distinctive nutritional deficiencies of communities. Future studies should standardize the dose–response relationships of interventions, conduct extended follow-up periods of at least twenty-four months, and conduct rigorous cost-effectiveness assessments to facilitate wide implementation and inform the uptake of evidence-based policies.

## Figures and Tables

**Table 1:** Inclusion and Exclusion Criteria for Study Selection.

No.	Item	Inclusion Criteria	Exclusion Criteria	Search Coherence
1	*Population*	Children recruited from schools or kindergartens (until 12 years old)	Children not recruited from preschools or primary schools. Children with medical attention by an illness diagnostic (treatment for cancer). Sport/athlete children not recruited from school (recruited form sport teams).	preschool* OR kindergarten OR school OR “elementary education” OR “primary education”
2	*Intervention/* *Exposure*	Children participating in a PA program with supplementation	Children not participating in PA (videogames, virtual reality). Children not receiving supplementation, in addition to PA program Intervention for parents. Children receiving supplements in order to address a certain illness. Programs with nutrition suggestion, but not supplementation. Supplementation affecting PA levels. Study protocols.	supplement* AND (intervention OR program*) AND (exercise OR “Physical activity” OR “physical education” OR sport OR fitness OR aerobic)
3	*Comparison*	-	-	-
4	*Outcome(s)*	Any	-	-
5	*Study Design*	Randomized controlled trials	Non-randomized controlled trials	“randomized controlled trial”
6	*Other Criteria*	Peer-reviewed, original, full-text studies	Non-peer-reviewed, non-original (systematic reviews, meta-analysis) or conference papers	-

**Table 2:** Methodological Quality Assessment of Included Studies Using the PEDro Checklist.

First Author, Year	PEDro Item										Score	Quality
	1	2	3	4	5	6	7	8	9	10		
Long et al. (2022) [29]	+	+	−	−	−	−	+	+	+	+	6/10	Good
Isdiany et al. (2021) [30]	+	−	+	−	−	−	+	−	+	+	5/10	Fair
Nqweniso et al. (2021) [31]	+	−	+	−	−	−	+	+	+	+	6/10	Good
Bass et al. (2007) [32]	+	−	+	−	−	+	+	−	+	+	6/10	Good
Beckmann et al. (2022) [33]	+	+	−	−	−	−	−	+	+	+	5/10	Fair
Teshome et al. (2024) [34]	+	+	+	−	−	−	+	+	+	+	7/10	Good
Ward et al. (2007) [35]	+	+	+	−	−	+	+	−	+	+	7/10	Good
Goodarzi and Hemayattalab (2012) [36]	+	−	+	−	−	−	−	−	+	+	4/10	Fair
Ianc et al. (2006) [37]	+	−	+	−	−	−	+	+	+	+	6/10	Good
Tse et al. (2023) [38]	+	+	+	−	−	−	−	−	+	+	5/10	Fair
French et al. (2005) [39]	+	−	+	−	−	−	+	−	+	+	5/10	Fair
Iuliano-Burns et al. (2003) [40]	+	−	+	−	−	−	+	−	+	+	5/10	Fair
Long et al. (2024) [41]	+	+	−	−	−	−	−	+	+	+	5/10	Fair

**Note.** 1: Random allocation; 2: Concealed allocation; 3: Baseline comparability; 4: Blind participants; 5: Blind therapists; 6: Blind assessors; 7: Adequate follow-up; 8: Intention-to-treat analysis; 9: Between-group comparisons; 10: Point estimates and variability.

**Table 3:** Risk of bias using RoB-2.

Study/Criteria	1	2	3	4	5	6	7
Long et al. (2022) [29]	** 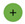 **	** 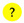 **	** 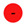 **	** 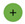 **	** 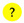 **	** 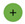 **	** 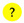 **
Isdiany et al. (2021) [30]	** 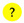 **	** 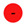 **	** 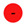 **	** 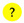 **	** 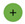 **	** 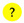 **	** 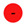 **
Nqweniso et al. (2021) [31]	** 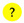 **	** 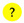 **	** 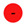 **	** 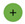 **	** 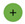 **	** 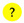 **	** 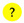 **
Bass et al. (2007) [32]	** 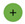 **	** 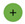 **	** 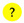 **	** 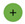 **	** 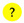 **	** 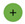 **	** 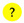 **
Beckmann et al. (2022) [33]	** 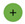 **	** 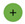 **	** 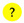 **	** 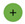 **	** 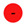 **	** 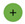 **	** 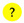 **
Teshome et al. (2024) [34]	** 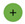 **	** 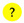 **	** 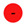 **	** 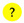 **	** 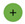 **	** 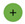 **	** 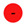 **
Ward et al. (2007) [35]	** 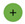 **	** 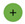 **	** 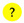 **	** 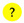 **	** 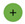 **	** 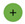 **	** 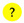 **
Goodarzi and Hemayattalab (2012) [36]	** 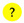 **	** 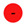 **	** 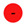 **	** 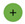 **	** 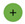 **	** 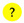 **	** 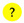 **
Ianc et al. (2006) [37]	** 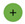 **	** 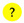 **	** 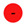 **	** 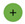 **	** 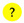 **	** 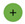 **	** 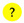 **
Tse et al. (2023) [38]	** 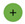 **	** 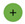 **	** 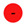 **	** 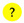 **	** 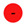 **	** 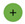 **	** 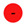 **
French et al. (2005) [39]	** 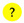 **	** 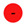 **	** 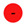 **	** 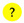 **	** 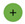 **	** 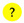 **	** 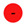 **
Iuliano-Burns et al. (2003) [40]	** 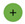 **	** 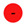 **	** 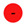 **	** 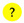 **	** 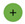 **	** 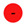 **	** 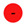 **
Long et al. (2024) [41]	** 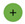 **	** 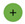 **	** 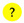 **	** 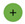 **	** 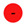 **	** 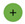 **	** 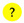 **

**Note.** 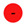: High risk; 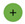: Low risk; 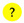: Some concerns; 1: Random sequence generation; 2: Allocation concealment; 3: Blinding of participants and personnel; 4. Blinding of outcome assessment; 5: Incomplete outcome data; 6: Selective reporting; 7: Other bias.

**Table 4:** Characteristics of included studies.

Authors	Sample Characteristics	Nutritional Intervention	Physical Exercise Intervention	Variables	Main Results	Key Aspects
Long et al. (2022) [29]	***N*** = 1304 (PA: 347; MMNS: 325; PA + MMNS: 297; Control: 335) ***Sex:*** 614 girls, 690 boys ***Age:*** 8.36 ± 0.40 years ***Country:*** South Africa ***Setting:*** Primary schools ***Pathologies:*** ~15% overweight/obese; ~38% stunted ***Dropouts:*** *n* = 77 (5.9%)	**Duration:** 36 weeks ***MMNS Group:*** Daily chewing tablet containing vitamins and trace elements based on MixMe™ powder (modified with 4500 mg β-carotene replacing vitamin A) ***PA + Control Groups:*** Placebo tablet with same packaging and similar taste ***PA + MMNS Group:*** Daily supplement + PA program	**Duration:** 36 weeks ***PA Group:*** Daily in-class activity breaks + 2 weekly sessions (45–60 min each): 1 session: Playful physical education lessons and 1 session: Dancing-to-music and improvised movements (Moving to Music) ***MMNS + Control Groups:*** Standard school curriculum	***Body composition*** Fat mass (FM) Fat free mass (FFM) Truncal fat mass (TrFM) Truncal fat free mass (TrFFM) ***Height velocity (HV)*** Stratification: <−2.8 cm vs. >−2.8 cm	***Main effects (adjusted models)*** **PA Group:** ↓ FM (*p* = 0.03) ↓ TrFM (*p* < 0.01) **MMNS Group** ↑ FFM (*p* < 0.01) ***Sex-specific effects (girls only)*** **PA Group**: ↓ FM (*p* = 0.02); ↓ TrFM (*p* = 0.02) **MMNS Group:** ↑ FFM (*p* = 0.03) ***Growth velocity interactions*** **PA × HV:** Children with lower HV showed ↓ FM (*B* = 0.12, *95% CI* = 0.003–0.237, *p* = 0.04) **MMNS × HV:** Children with lower HV showed ↑ FFM (*B* = 0.30, *95% CI* = 0.25–0.42, *p* = 0.01) **Both PA and MMNS:** Children with lower HV had ↓ TrFM vs. controls (*p* = 0.01 for both)	PA reduced fat mass while MMNS increased fat-free mass, particularly in girls and slow-growing children. Children with slower height velocity showed greater body composition benefits from both interventions. School-based PA sessions plus daily micronutrient supplementation effectively address malnutrition and obesity prevention.
Isdiany et al. [30]	***N*** = 30 (Treatment: 15; Control: 15) ***Sex:*** 13 boys, 17 girls ***Age:*** 10.23 ± 1.56 years ***Country:*** Indonesia ***Setting:*** Primary school ***Pathologies:*** Stunted (H/A z-score <−2 SD) ***Dropouts:*** NR	**Duration:** 3 months ***Treatment Group (TG):*** 5 mL zinc syrup (20 mg zinc sulfate monohydrate) 3 times/week + physical exercise ***Control Group (CG):*** No zinc supplementation	**Duration:** 3 months ***TG + CG:*** Physical fitness exercise 3 times/week using video guidance, monitored via WhatsApp Group ***TG:*** Physical fitness for elementary school students (not age-differentiated)	***Height*** Absolute height Height-for-age z-score (H/A z-score) ***Academic performance*** Average scores from Mathematics and Indonesian subjects	***Height changes*** **TG:** ↑ 2.10 cm (121.6 → 123.7 cm, *p* < 0.05) **CG:** ↑ 1.72 cm (125.2 → 126.9 cm, *p* < 0.05) **Between groups:** ND (*p* > 0.05) ***H/A z-score changes*** **TG:** ↑ 0.19 (−2.62 → −2.43, *p* < 0.05) **CG:** ↑ 0.14 (−2.43 → −2.29, *p* < 0.05) **Between groups:** ND (*p* > 0.05) ***Academic performance*** **TG:** 0.79 (80.20 → 79.41, *p* > 0.05) **CG:** 0.35 (79.58 → 79.93, *p* > 0.05) **Between groups:** ND (*p* > 0.05)	Three-month zinc supplementation plus exercise showed no benefits over exercise alone. Home-based video exercise reduced intervention effectiveness versus supervised. Minimum 6-month intervention needed for meaningful zinc supplementation effects.
Nqweniso et al. [31]	***N*** = 898 ***Sex:*** 458 boys, 440 girls ***Age:*** 8–11 years ***Country:*** South Africa ***Setting:*** 8 quintile 3 schools in Gqeberha ***Pathologies:*** Double burden of malnutrition ***Dropouts:*** *n* = 111 (11.0%)	**Duration:** 10 weeks ***E3 Group:*** Health and hygiene education + nutrition education with Ready-to-Use Supplementary Food (RUSF) once daily (530 kcal/100 g sachet) ***E4 Group:*** PA + health/hygiene education + nutrition education with RUSF ***Control Groups:*** Standard school curriculum + deworming medication	**Duration:** 10 weeks ***E1 Group:*** PA only—2 weekly PE lessons (40 min) + 1 weekly moving-to-music lesson (40 min) + regular in-class PA breaks + playground adaptations ***E2 Group:*** PA + health and hygiene education (same PA as E1) ***Control Groups:*** Standard school curriculum	***Body composition*** BMI BMI-for-age z-scores Body fat percentage (BF%) via skinfold measurements (triceps and subscapular)	***Total sample changes*** ↑ BMI (17.0 → 17.7 kg/m^2^, *p* < 0.001) ↑ BMI-for-age (−0.0 → 0.1, *p* < 0.001) ↑ BF% (15.9 → 17.2%, *p* < 0.001) ***Intervention effects by nutritional status:* Normal-weight children:** PA interventions (alone or with health education) mitigated BF% increases compared to controls **Overweight/obese children:** PA intervention showed beneficial effects on BF%, but not when combined with health education **Combined interventions:** E4 group showed increased BMI-for-age compared to controls	PA mitigated body fat increases, particularly in normal-weight children. Normal-weight children benefited more from interventions than overweight/obese peers. Combined interventions showed unexpected BMI increases versus single approaches.
Bass et al. [32]	***N*** = 88 (pre- and early-pubertal boys) ***Sex:*** Boys ***Age:*** 7–11 years (mean 9.0 ± 0.2 years) ***Country:*** Australia ***Setting:*** School-based intervention ***Pathologies:*** NR	**Duration:** 34 weeks ***Ca Groups:*** Calcium-fortified foods using milk minerals (392 ± 29 mg/day additional calcium) ***Placebo Groups:*** Same food products without added calcium ***Food products:*** 10 varieties of muffins and cookies (one product per day, seven per week)	**Duration:** 34 weeks ***Exercise Groups:*** Moderate-impact exercise—20 min min, 3 times/week including hopping, jumping, skipping (ground reaction forces 2–8 times body weight) ***No-Exercise Groups:*** Low-impact exercise—20 min, 3 times/week including stretching, low-impact games (~1 body weight)	***Bone health outcomes*** Bone mineral content (BMC) at loaded sites: femur, tibia-fibula BMC at non-loaded sites: humerus, radius-ulna, lumbar spine ***Body composition*** Lean mass Fat mass	***Loaded sites (femur)*** **Ex + Ca group:** 2% greater BMC increase than Ex + placebo, No-Ex + Ca, or No-Ex + placebo groups (all *p* < 0.03) ***Loaded sites (tibia-fibula)*** **Ex + Ca group:** 3% greater BMC increase than No-Ex + placebo (*p* < 0.02); 2% greater than Ex + placebo and No-Ex + Ca (NS) ***Non-loaded sites:*** No significant effects at humerus, radius-ulna, or lumbar spine ***Exercise trends:*** 1.1% greater femur BMC increase in Ex vs. No-Ex groups (*p* = 0.056) ***Calcium trends:*** 1.1% greater femur BMC increase in calcium vs. placebo groups (*p* = 0.06)	Combined exercise and calcium supplementation produced 2–3% greater BMC increases at loaded sites than either intervention alone. Benefits were limited to mechanically loaded skeletal sites (femur, tibia-fibula) with no effects at non-loaded sites. Even in boys with adequate dietary calcium intakes, additional supplementation enhanced exercise-induced bone benefits.
Beckmann et al. [33]	***N*** = 932 ***Sex:*** 458 girls, 474 boys ***Age:*** 8.42 ± 1.94 years (6–12 years) ***Country:*** South Africa ***Setting:*** 4 quintile-3 public primary schools ***Pathologies:*** Disadvantaged schools, some stunting (~9.5%), overweight/obesity (~17%) ***Dropouts:*** *n* = 433 (31.7%)	**Duration:** 12 weeks (actual intervention interrupted by 3-week school holiday) ***MMNS Groups:*** Daily orange-flavored chewing tablet containing multi-micronutrients (formulated based on MixMe™ powder, modified with DSM Nutritional Products) ***Placebo Groups:*** Daily placebo tablet with similar taste/appearance.	**Duration:** 12 weeks ***PA Groups:*** 2 weekly 45 min sessions: 1 “moving to music” lesson, and 1 “physical education” lesson Based on KaziKidz toolkit, assisted by physical education coach ***Non-PA Groups:*** Standard school curriculum	***Cognitive performance*** Information processing (congruent trials—Flanker task) Inhibitory control (incongruent trials—Flanker task) Reaction time and accuracy measures ***Academic success*** End-of-year results (mean of home language and maths)	***Cognitive performance*** **All groups:** ↓ Reaction time and accuracy for congruent and incongruent trials (*p* < 0.05) **No significant group × time interactions** for any cognitive measures ***Academic achievement*** **PA + MMNS vs. MMNS:** Combined intervention showed higher academic achievement than MMNS alone (*p* < 0.001) **No significant differences** between combined intervention vs. PA alone or vs. placebo **Single interventions:** Both PA and MMNS groups showed academic decline, while combined and placebo groups improved	Combined PA and MMNS intervention showed no additional cognitive performance compared to placebo. Only the combined intervention showed superior academic achievement compared to MMNS alone, with no benefits versus PA alone or placebo. School holiday interruptions and possible ceiling effects in cognitive performance may have limited intervention effectiveness in this population.
Teshome et al. [34]	***N*** = 69 ***Sex:*** 31 boys, 38 girls ***Age:*** 5–7 years ***Country:*** Ethiopia ***Setting:*** 3 quintile schools (kindergartens and primary schools) ***Pathologies:*** Moderate thinness (BMI-for-age ≥−3 to <−2) ***Dropouts:*** *n* = 6 (8.0%)	**Duration:** 12 weeks ***RUSF Groups:*** Ready-to-use supplementary food—500 kcal/day (12.5 g proteins, 31g fat, 42.8 g carbohydrates), 7 sachets per week ***Control Group:*** No dietary intervention	**Duration:** 12 weeks ***HiML Groups:*** High-intensity motor learning training—60 min/ day, 5 days/week combined active play activities (hopping, jumping, skipping, ball skills) with training/rest ratio 70–30% ***Non-HiML Groups:*** No PA intervention	***Motor skill-related physical fitness*** PERF-FIT test battery: Stepping, side jump, standing long jump, overhand throw Bounce and catch, throw and catch, static/dynamic balance, jumping and hopping	**RUSF Group:** ↑ Stepping ↑ Side jump ↑ Standing long jump ↑ Bounce and catch ↑ Throw and catch ↑ Jumping and hopping **RUSF + HiML Group:** ↑ Stepping ↑ Side jump ↑ Standing long jump ↑ Bounce and catch ↑ Throw and catch ↑ Jumping and hopping **Control Group:** ND **RUSF + HiML vs. RUSF:** Superior improvements in side jump, bounce and catch, throw and catch	RUSF + HiML showed greatest improvements in ball skills (bounce/catch, throw/catch) compared to RUSF alone or control. Both RUSF groups (with/without HiML) showed similar improvements in basic motor fitness compared to control. HiML training provided additional benefits specifically for coordination-demanding tasks requiring motor skill learning.
Ward et al. [35]	***N*** = 75 ***Sex:*** 27 boys, 48 girls ***Age:*** 9.8–10.8 years ***Country:*** United Kingdom ***Setting:*** Primary schools and gymnastics clubs ***Pathologies:*** Healthy pre-pubertal children ***Dropouts:*** *n* = 11 (12.8%)	**Duration:** 48 weeks ***Calcium Groups:*** 500 mg elemental calcium daily (1250 mg calcium carbonate salt—Calcichew™) ***Placebo Groups:*** Identical placebo tablets (same shape, taste, texture without calcium)	**Duration:** 48 weeks ***Gymnast Groups:*** Elite gymnasts (>10 h/week training, loads up to 20× body weight) ***Control Groups:*** School children (standard PA)	***Bone parameters*** *pQCT:* Trabecular and cortical vBMD at distal radius and tibia; cortical vBMD at midshaft *DXA:* Lumbar spine BMAD, BMC; whole body BMC, bone area, lean/fat mass *Bone geometry:* Cross-sectional area, cortical area, cortical thickness, SSI	**Controls + Calcium (*p* < 0.05)** ↑ Tibia trabecular vBMD (5% increase) ↑ Muscle area at tibia (3%) ↑ Whole body fat mass (14%) **Gymnasts:** ND **Calcium-exercise interaction (*p* = 0.04):** Controls responded more than gymnasts to calcium supplementation for tibia trabecular vBMD (1.05 vs. 0.98 ratio) **No beneficial effects** of additional calcium in gymnasts who already consumed adequate calcium intake (888 mg/day vs. UK RNI of 555–800 mg/day)	Controls benefited more from calcium supplementation than elite gymnasts, contrary to the study’s primary hypothesis. Gymnasts already consuming recommended calcium showed no additional benefit from supplementation. High-intensity exercise (gymnastics) may optimize skeletal adaptation, reducing the capacity for further calcium-induced improvements.
Goodarzi and Hemayattalab [36]	***N*** = 60 ***Sex:*** Boys ***Age:*** 8-10 years ***Country:*** Iran ***Setting:*** Special schools ***Pathologies:*** Autism spectrum disorders ***Dropouts:*** NR	**Duration:** 24 weeks ***Calcium Groups:*** 2000 cc enriched cow milk with vitamin D providing 250 mg additional calcium/day ***Control Groups:*** No calcium supplementation	**Duration:** 24 weeks ***Exercise Groups:*** Weight bearing exercise 50 min, 3 sessions/week including walking, running, jumping, hopping, and galloping ***Non-Exercise Groups:*** No structured exercise intervention	***Bone parameters*** Femoral neck BMD (g/cm^2^) measured by dual-energy X-ray absorptiometry	**Ex+Ca+ Group:** ↑ Femoral neck BMD (0.625 to 0.643 g/cm^2^, +18.75% greater than control) **Ex+Ca−** **Group:** ↑ Femoral neck BMD (0.625 to 0.633 g/cm^2^, +12.21%) **Ex–Ca+ Group:** ↑ Femoral neck BMD (0.624 to 0.628 g/cm^2^, +7.5% increase) **Ex–Ca− Group:** No significant change. **Combined intervention (*p* < 0.05):** Ex+Ca+ group had 14.04% greater than Ex+Ca− group and 18.75% greater than Ca+ alone. **Exercise vs. Calcium (*p* < 0.05):** Exercise effect was greater than calcium (Ex+Ca− achieved 4.71% greater BMD than Ex−Ca+ group) **All experimental groups** had significantly greater BMD than control group (*p* < 0.05)	Combined exercise and calcium supplementation (22.68% increase) was more effective than either intervention alone in children with autism. Weight-bearing exercise showed greater osteogenic effects than calcium supplementation alone in this population.
Ianc et al. [37]	***N*** = 153 (Sp+Ca+: 38; Sp+Ca−: 39; Sp−Ca+: 36; Sp−Ca−: 40) ***Sex:*** 74 girls, 79 boys ***Age:*** 8–11 years (mean ~9.6 years) ***Country:*** Romania ***Setting:*** Local schools ***Pathologies:*** None (healthy children, sedentary) ***Dropouts:*** *n* = 7 (4.4%)	**Duration:** 24 weeks ***Calcium Group:*** Daily 800 mg calcium-phosphate powder extracted from milk ***Placebo Group:*** Lactose powder with identical packaging ***Compliance:*** Assessed monthly through powder bag returns; <75% compliance = withdrawal	**Duration:** 6 weeks ***Active Group:*** 50 min, twice weekly additional sessions beyond standard PE classes (10 min warm-up + 30 min workout with lower limb strengthening, high-impact games, plyometric jumps, gymnastics + 10 min cool-down) ***Nonactive Group:*** Standard school curriculum	***Bone ultrasound*** Ad-SoS (amplitude-dependent speed of sound) at phalanx UBPI (ultrasound bone profile index) ***Bone architecture*** Hmean parameter (fractal analysis of calcaneus radiographs) ***Anthropometric measures*** Height, weight, BMI, body composition	***Calcium-specific effects*** **Calcium Group** ↑ Ad-SoS vs. placebo (*p* = 0.01) in complier cohort ↑ UBPI vs. placebo (*p* < 0.05) in complier cohort ***Exercise-specific effects*** **Active Group** ↑ Hmean vs. nonactive (*p* < 0.05) in both intention-to-treat and complier cohorts ***Combined effects*** **Sp+Ca+ Group** Greatest Hmean gain, significantly higher than Sp−Ca− group (*p* < 0.05)	Calcium supplementation had systemic effects on bone ultrasound properties (cortical bone), while exercise specifically improved trabecular microarchitecture at weight-bearing sites. Statistical interaction between calcium and exercise confirmed differential and synergistic effects on bone tissue.
Tse et al. [38]	***N*** = 62 (Cycling: 18; Melatonin: 14; Combination: 12; Placebo: 18) ***Sex:*** 50 boys, 12 girls ***Age:*** 8–12 years (mean ~9.9 years) ***Country:*** China ***Setting:*** Special schools ***Pathologies:*** Autism Spectrum Disorder (ASD) ***Dropouts:*** *n* = 18 (22.5%)	**Duration:** 2 weeks ***Melatonin Group:*** 3 mg liquid melatonin (Natrol^®^) nightly, 30 min before bedtime ***Combination Group:*** melatonin dosage + cycling program ***Cycling + Placebo Groups:*** Inert liquid (similarly flavored water) ***Acclimation period:*** 2 weeks prior with placebo liquid for familiarization	**Duration:** 2 weeks ***Cycling Group:*** 10 sessions (5 week, 60 min) outdoor bicycle training with 1:1 instructor supervision, progressively distance and intensity, RPE 3–5 (OMNI scale) ***Combination Group:*** Cycling program + melatonin ***Control Groups:*** Standard daily routine, no add PA	***Sleep parameters (actigraphy)*** Sleep efficiency (SE) Sleep onset latency (SOL) Sleep duration (SDur) Wake after sleep onset (WASO) ***Sleep parameters (sleep log)*** Parent-reported sleep measures	***All intervention groups* vs. *placebo*** **Actigraphy results:** Significant improvements in SE, WASO, and SDur (all *p* < 0.05) with moderate-to-strong effect sizes (*d* = 0.52–0.98) **Sleep log results:** Significant improvements in SE and SOL (all *p* < 0.001) with large effect sizes (*d* = 1.08–1.91) ***Between-group comparisons*** **No significant differences** among the three intervention groups for any sleep parameters at both timepoints (*p* > 0.05) **Placebo group:** NS in any sleep parameters	All three interventions (cycling, melatonin, combination) showed similar effectiveness in improving sleep quality in ASD children. No additional benefits combining cycling and melatonin compared to either intervention alone. Cycling training increase melatonin production, similar effect to melatonin supplementation. Short intervention period improved across sleep parameters.
French et al. [39]	***N*** = 322 (Intervention: 15; Control: 15 troops) ***Sex:*** Girls only ***Age:*** 9–11 years (mean 10.5 years) ***Country:*** USA ***Setting:*** Girl Scout troops ***Pathologies:*** Healthy girls ***Dropouts:*** Individual retention 92% (296/322 completed all visits)	**Duration:** 92 weeks ***Intervention Group:*** Behavioral program targeting 1300 mg/day calcium intake (800 mg increase through 4 additional daily servings of calcium-rich foods) via troop activities, web-based training, and summer camp. Baseline intake already high at 1265 mg/day. ***Control Group:*** Standard troop activities, no dietary intervention.	**Duration:** 92 weeks ***Intervention Group:*** 120 min/week weight-bearing PA using Social Cognitive Theory. Delivered through 10 annual troop sessions (90 min each), web-based program, and summer camp with goal-setting, self-monitoring, and incentives. ***Control Group:*** Standard troop activities, no PA intervention.	***Bone parameters*** Bone mineral content (BMC), density (BMD) and area (BA) by at total body, lumbar spine (L1–L4), proximal femur, femoral neck, and one-third distal radius ***Healthy habits*** Dietary calcium intake (24 h recall) Weight-bearing PA (PACI)	***Bone outcomes*** **No significant intervention effects** for BMC at any bone site (total body, total hip, femoral neck, or 1/3 distal radius) (*p* > 0.05) ***Healthy habits*** **Calcium intake:** Significant increase in intervention vs. control groups (*p* < 0.05), but both groups remained at recommended levels throughout study **PA:** No significant intervention effects for WBPA (*p* > 0.05)	Community-based behavioral intervention was ineffective for increasing bone mass gains or PA. Significant increases in dietary calcium intake occurred, but baseline levels were already at recommended levels. High-quality study design with excellent retention (92%) but null results suggest need for more structured interventions.
Iuliano-Burns et al. [40]	***N*** = 66 (Exercise + Calcium: 16; Exercise + Placebo: 18; Non-exercise + Calcium: 14; Non-exercise + Placebo: 18) ***Sex:*** Girls only ***Age:*** 7–11 years (mean 8.8 ± 0.1 years) ***Country:*** Australia ***Setting:*** School-based ***Pathologies:*** Pre- and early-pubertal girls (80% Tanner Stage 1, 20% Tanner Stage 2), 15% Asian descent ***Dropouts:*** *n* = 9 (12%)	**Duration:** 34 weeks ***Calcium Group:*** Ca-fortified foods containing 434 ± 19 mg/day calcium from milk minerals (400 mg calcium from 2 g milk minerals) ***Food products:*** 10 items weekly from 25 varieties of muffins, cookies, and muesli bars ***Total calcium intake:*** Increased from 673 ± 35 to 1121 ± 45 mg/day ***Placebo Group:*** Same foods without added calcium (equivalent basic mixture instead) ***Compliance:*** 70% in both groups	**Duration:** 34 weeks ***Moderate-impact Exercise:*** 20 min, 3 times/week during PE classes (Hopping, jumping, and skipping-based activities) producing 2–4 times body weight ground reaction forces. ***Low-impact Exercise (Control):*** Same schedule but activities producing ≤1 body weight (stretching, low-impact dance).	***Bone parameters*** Bone mineral content (BMC) measured by DXA at total body, lumbar spine, leg (femur, tibia-fibula), and arm (humerus, radius-ulna) ***Anthropometric measures*** Body composition, anthropometry, sexual maturation ***Healthy habits*** PA, and dietary intake	***Exercise-calcium interaction effects*** **Femur:** Significant interaction (7.1%, *p* < 0.05) − exercise + calcium produced greater benefits than either intervention alone ***Main effects at loaded sites*** **Tibia-fibula:** Exercise main effect (3% greater increase, *p* < 0.05) but no calcium effect or interaction ***Main effects at non-loaded sites*** **Humerus:** Calcium main effect (12.0% vs. 9.8%, *p* = 0.09) **Radius-ulna:** Calcium main effect (12.6% vs. 8.6%, *p* < 0.01) ***No effects detected*** **Lumbar spine:** No exercise or calcium effects for BMC, height, area, or volume	Regional specificity demonstrated: exercise + calcium interaction at loaded sites (femur), exercise-only effects at loaded sites (tibia-fibula), calcium-only effects at non-loaded sites (arms). Combining moderate exercise with calcium supplementation produces additive/multiplicative effects at mechanically loaded skeletal sites. Short-duration study (8.5 months) with relatively low calcium supplementation dose but significant site-specific bone mass gains.
Long et al. (2024) [41]	***N*** = 1304 children from 2019 to 2021 (PA: 347; MMNS: 325; PA + MMNS: 297; Control: 335) ***Sex:*** 637 girls, 667 boys ***Age:*** 6–12 years (mean ~8.36 ± 0.40 years) ***Country:*** South Africa ***Setting:*** Quintile 3 public schools in periurban marginalized communities ***Pathologies:*** ~15% overweight/obese; ~38% stunted ***Dropouts:*** *n* = 77 (5.9%)	**Duration:** 36 weeks ***MMNS Group:*** Daily chewing tablet containing vitamins and trace elements based on MixMe™ powder (modified with 4500 mg β-carotene replacing vitamin A) ***PA + Control Groups:*** Placebo tablet with same packaging and similar taste ***PA + MMNS Group:*** Daily supplement + PA program	**Duration:** 36 weeks ***PA Group:*** Daily in-class activity breaks + 2 weekly sessions (45–60 min each): 1 session: Playful physical education lessons and 1 session: Dancing-to-music and improvised movements (Moving to Music) ***MMNS + Control Groups:*** Standard school curriculum	***Body composition*** Fat mass (FM) Fat free mass (FFM) Truncal fat mass (TrFM) Truncal fat free mass (TrFFM) ***Height velocity (HV)*** Stratification: <−2.8 cm vs. >−2.8 cm	***Main effects (adjusted models)*** **PA Group:** ↓ FM (*p* = 0.03) ↓ TrFM (*p* < 0.01) **MMNS Group:** ↑ FFM (*p* < 0.01) ***Sex-specific effects (girls only)*** **PA Group**: ↓ FM (*p* = 0.02); ↓ TrFM (*p* = 0.02) **MMNS Group:** ↑ FFM (*p* = 0.03) ***Growth velocity interactions*** **PA × HV:** Children with lower HV showed ↓ FM (*B* = 0.12, *95% CI* = 0.003–0.237, *p* = 0.04) **MMNS × HV:** Children with lower HV showed ↑ FFM (*B* = 0.30, *95% CI* = 0.25–0.42, *p* = 0.01) **Both PA and MMNS:** Children with lower HV had ↓ TrFM vs. controls (*p* = 0.01 for both)	PA reduced fat mass while MMNS increased fat-free mass, particularly in girls and slow-growing children. Children with slower height velocity showed greater body composition benefits from both interventions. School-based PA sessions plus daily micronutrient supplementation effectively address malnutrition and obesity prevention.

**Note.** PA = Physical Activity; MMNS = Multi-Micronutrient Supplementation; BMI = Body Mass Index; BMC = Bone Mineral Content; BMD = Bone Mineral Density; vBMD = volumetric Bone Mineral Density; FM = Fat Mass; FFM = Fat Free Mass; TrFM = Truncal Fat Mass; TrFFM = Truncal Fat Free Mass; HV = Height Velocity; BF% = Body Fat Percentage; pQCT = peripheral Quantitative Computed Tomography; DXA = Dual-energy X-ray Absorptiometry; BMAD = Bone Mineral Apparent Density; SSI = Stress Strain Index; RUSF = Ready-to-Use Supplementary Food; HiML = High-intensity Motor Learning; ASD = Autism Spectrum Disorder; SE = Sleep Efficiency; SOL = Sleep Onset Latency; SDur = Sleep Duration; WASO = Wake After Sleep Onset; RPE = Rating of Perceived Exertion; PACI = Physical Activity Checklist Interview; WBPA = Weight-Bearing Physical Activity; Ad-SoS = Amplitude-dependent Speed of Sound; UBPI = Ultrasound Bone Profile Index. ↑ indicates significant increase; ↓ indicates significant decrease; ND = No difference; NS = Not significant; NR = Not reported. All *p*-values represent statistical significance with *p* < 0.05 unless otherwise specified. Sample sizes reflect baseline enrollment; dropout rates are reported where available.
